# An exosome-based liquid biopsy signature for pre-operative identification of lymph node metastasis in patients with pathological high-risk T1 colorectal cancer

**DOI:** 10.1186/s12943-022-01685-8

**Published:** 2023-01-06

**Authors:** Katsuki Miyazaki, Yuma Wada, Keisuke Okuno, Tatsuro Murano, Yuji Morine, Tetsuya Ikemoto, Yu Saito, Hiroaki Ikematsu, Yusuke Kinugasa, Mitsuo Shimada, Ajay Goel

**Affiliations:** 1 Department of Molecular Diagnostics and Experimental Therapeutics, Beckman Research Institute of City of Hope, Biomedical Research Center, 1218 S. Fifth Avenue, Suite 2226, Monrovia, CA 91016 USA; 2 Department of Surgery, Tokushima University, Tokushima, Japan; 3 Department of Gastrointestinal Surgery, Tokyo Medical and Dental University, Tokyo, Japan; 4 Department of Gastroenterology and Endoscopy, National Cancer Center Hospital East, Chiba, Japan; 5 City of Hope Comprehensive Cancer Center, Duarte, CA USA

**Keywords:** Exosomal miRNA, Cell-free miRNA, T1 CRC, Lymph node metastasis, Liquid biopsy

## Abstract

**Background:**

According to current guidelines, more than 70% of patients with invasive submucosal colorectal cancer (T1 CRC) undergo a radical operation with lymph node dissection, even though only ~ 10% have lymph node metastasis (LNM). Hence, there is imperative to develop biomarkers that can help robustly identify LNM-positive patients to prevent such overtreatments. Given the emerging interest in exosomal cargo as a source for biomarker development in cancer, we examined the potential of exosomal miRNAs as LNM prediction biomarkers in T1 CRC.

**Methods:**

We analyzed 200 patients with high-risk T1 CRC from two independent cohorts, including a training (*n* = 58) and a validation cohort (*n* = 142). Cell-free and exosomal RNAs from pre-operative serum were extracted, followed by quantitative reverse-transcription polymerase chain reactions for a panel of miRNAs.

**Results:**

A panel of four miRNAs (miR-181b, miR-193b, miR-195, and miR-411) exhibited robust ability for detecting LNM in the exosomal vs. cell-free component. We subsequently established a cell-free and exosomal combination signature, successfully validated in two independent clinical cohorts (AUC, 0.84; 95% CI 0.70–0.98). Finally, we developed a risk-stratification model by including key pathological features, which reduced the false positive rates for LNM by 76% without missing any true LNM-positive patients.

**Conclusions:**

Our novel exosomal miRNA-based liquid biopsy signature robustly identifies T1 CRC patients at risk of LNM in a preoperative setting. This could be clinically transformative in reducing the significant overtreatment burden of this malignancy.

**Supplementary Information:**

The online version contains supplementary material available at 10.1186/s12943-022-01685-8.

By virtue of the advances made in endoscopic techniques, many of the patients with invasive submucosal colorectal cancers (T1 CRCs) who previously required surgery can now be effectively treated with endoscopic resections – either by endoscopic mucosal resection (EMR) or via endoscopic submucosal dissection (ESD) [1]. Endoscopic approaches can not only achieve curative resection of T1 CRCs [1, 2], but are less invasive, associated with lower morbidity rates, shorter hospital stays [2, 3], and less expensive compared to surgical resection [4]. T1 CRC patients with submucosal invasion depth of > 1000 μm, positive lymphovascular invasion, poorly differentiated tumors, and high tumor budding grade are deemed as critical risk factors for lymph node metastasis (LNM) – and any patient exhibiting one or more of these risk factors in endoscopically resected specimens are recommended additional surgical operation [5–7]. However, these current pathologic factors for the risk stratification of LNM-positive T1 CRCs are inadequate, primarily because of their low positive predictive value (PPV). Interestingly, an overwhelming majority of studies have repeatedly demonstrated that only about 10–15% of T1 CRC patients deemed as high-risk based upon current pathological features have LNM based upon the examination of post-surgery resected specimens [1, 6, 7]. These studies highlight that radical surgeries currently result in the overtreatment of as much as 70% of patients with T1 CRCs. Therefore, it is imperative to develop more accurate biomarkers to help robustly identify LNM-positive patients who are genuinely high-risk and spare the majority for management with endoscopic resections.

In recent years, exosomes and extracellular vesicles have received increasing attention as promising cancer biomarkers in liquid biopsy settings [8, 9]. While exosomal miRNAs (exo-miRNAs) are considered as one the most abundant and stable molecules within exosomes [8], it remains unclear whether these might offer superior cancer specificity vis-à-vis cell-free miRNAs (cf-miRNAs) that are highly sensitive but lack tumor specificity [10]. Accordingly, we hypothesized that combining cf- and exo-miRNAs might offer an optimal combination of sensitivity and specificity required for cancer patients. We have recently provided early evidence supporting this hypothesis, where we demonstrated that combining these two types of miRNA markers yielded superior diagnostic performance in patients with pancreatic ductal adenocarcinoma [11].

We have previously reported that a panel of 5 miRNAs allowed robust detection of LNM in tissue specimens of patients with T1 CRC [12]. Subsequently, we validated the performance of these biomarkers in a liquid biopsy assay, which yielded a reduced panel of 4 cf-miRNAs for identifying LNM in patients with T1 CRC [13]. Finally, in this study, we developed a signature by combining the analysis of cf- and exo-miRNAs, which showed superior diagnostic accuracy. We highlighted its clinical significance for predicting LNM in patients suffering from this lethal malignancy.

## Materials and methods

This retrospective cohort study included a total of 200 high-risk T1 CRC patients who were subjected to radical surgery. These patients were enrolled at two independent institutions. Pathological high-risk LNM patients were diagnosed according to the Japanese Society for Cancer of the Colon and Rectum guidelines 2019 for treating colorectal cancer. Pre-operative serum samples were obtained from these patients and were used for analysis in this study.

For the extraction of cf- and exo-RNA, 200 μL of serum was used for each. Total exosome isolation was performed using the Total Exosome Isolation Kit (ThermoFisher Scientific, Waltham, MA, USA). Qiagen miRNeasy Kit (Qiagen, Hilden, Germany) and TaqMan microRNA Reverse Transcription Kit (ThermoFisher Scientific, Waltham, MA, USA) were used for RNA purification followed by complementary DNA synthesis. Real-time reverse transcription quantitative PCR analysis was performed using the QuantStudio 7 Flex Real-Time PCR System (Applied Biosystems, Foster City, CA), and the expression of the target miRNAs was normalized to that of miR-16.

More detailed information on methods is provided in the Supplemental materials and methods.

## Results and discussion

### Development of a cell-free and exosomal miRNA combination panel for the identification of LNM in patients with T1 CRC

This retrospective cohort study included a total of 200 pathologically characterized high-risk T1 CRC patients who were subjected to radical surgery. These patients were enrolled at two independent institutions. The first cohort was used for biomarker training. It included 58 patients, with seven who were LNM-positive (LNP) and 51 LNM-negative (LNN) – enrolled at the Tokyo Medical and Dental University Hospital, Japan. The second cohort was assigned as the validation cohort and comprised 142 patients with 12 LNP and 130 LNN enrolled at the National Cancer Center Hospital East, Japan. Both clinical cohorts were considered clinicopathologically comparable, except for differences in age distribution and depth of tumor invasion, as illustrated in Supplemental Table 1. Pre-operative serum samples were obtained from these patients and were used for analysis in this study.

**Fig. 1:**
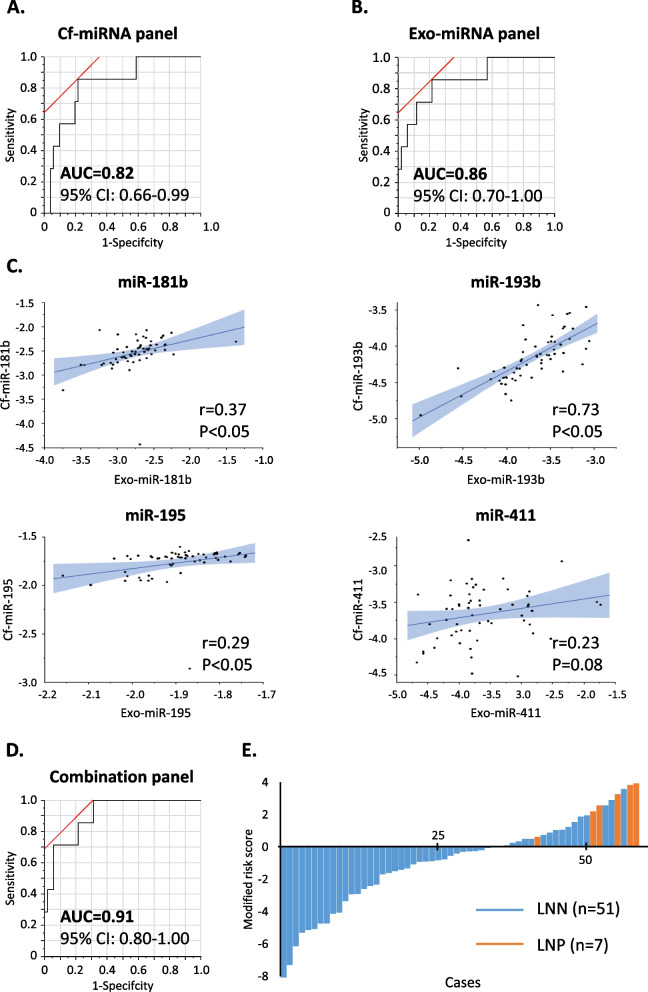
Training of exosomal miRNAs and cell-free miRNAs in predicting lymph node metastasis from high-risk T1 CRC patients. **A** A receiver operating characteristic (ROC) curve analysis to evaluate the performance of cell-free miRNAs panel. **B** ROC curve analysis to evaluate the performance of the exosomal miRNAs panel. **C** Correlation of 4 miRNAs’ expression between exosomal component and total serum. **D** ROC curve analysis to evaluate the performance of exosomal and cell-free miRNAs combination panel. **E** Waterfall plot for modified risk score distribution in a training cohort. Cf-: cell-free, exo: exosomal, LNN: lymph node metastasis negative, LNP: lymph node metastasis positive, AUC: area under the curve

Our previous studies identified a panel of 4 miRNAs, including miR-181b, miR-193b, miR-195, and miR-411, that could detect LNM in patients with T1 CRC. In this study, this panel of miRNA biomarkers, by their tumor-specificity within exosomal cargo, might offer superior diagnostic accuracy, individually or in combination with the analysis within the cell-free compartment. Therefore, we first analyzed the diagnostic performance of individual miRNAs for their ability to detect LNM in T1 CRC patients. As illustrated in Supplemental Fig. 1A and B, the ROC curves depict the AUC values of each miRNA in total cell-free serum (cf-miRNAs) and exosomal component (exo-miRNAs) within the training cohort. As illustrated in this figure, compared to cf-miRNAs, the diagnostic performance of exo-miRNAs was significantly superior for each of the individual markers, including miR-181b (AUC; 0.64 vs. 0.70), miR-193b (AUC; 0.61 vs. 0.69), miR-195 (AUC; 0.77 vs. 0.79), and miR-411 (AUC; 0.63 vs. 0.80). More importantly, it was interesting to witness that the combined analyses of all 4 miRNAs in both total serum and exosomes exhibited a significantly superior diagnostic performance to identify LNM in patients with T1 CRC (Fig. 1A, B), compared to individual miRNA markers. Furthermore, in support of our original hypothesis, in comparison to the cf-miRNA panel (AUC; 0.82, Fig. 1A), the exo-miRNA panel performed markedly better (AUC; 0.86, Fig. 1B).

Next, to determine the expression patterns of the individual miRNAs in total serum and exosomes, we performed a correlation analysis for each of the biomarkers between these two components (Fig. 1C). Interestingly, other than miR-193b (r; 0.73), the remaining 3 miRNAs revealed a weak correlation between the two components, miR-181b (r; 0.37), miR-195 (r; 0.29), and miR-411 (r; 0.23). These results support our hypothesis that since both types of miRNA markers are derived from different sources (e.g., necrotic, apoptotic, and circulating tumor cells in serum vs. exosomes), the overall diagnostic performance of a given assay might be more robust if one were to use the combination of the two for the detection of LNM in patients with T1 CRC.

Accordingly, we developed a combined panel of cf- and exo-miRNAs, which exhibited a significantly superior ability for LNM detection vis-à-vis individual marker panels (AUC; 0.91, 95% CI; 0.80–1.00; Fig. 1D). To further highlight the clinical significance of this combination panel, we noted that it possessed two times higher positive predictive value (PPV; 0.30) vs. current pathological guidelines (PPV; 0.12), supporting the rationale of a liquid biopsy assay based upon analysis of such miRNA markers can be of clinical significance in reducing the burden of surgical overtreatment in T1 CRC patients.

The waterfall plot derived from the modified risk scores using this combination panel further highlighted the clinical potential of these biomarkers. It revealed that all LNP patients within the training cohort could be classified at high risk for LNM (sensitivity; 1.00, Fig. 1E). We observed that both cf- and exo-miRNA panels had a sensitivity of 0.86, and in each panel, there was a single false negative case (different in each panel); however, when we used the combination panel, such cases were correctly classified, underscoring the biological and clinical significance for using such a combined biomarker panel that complemented each other’s potential.

### Successful validation of combination panel for predicting LNM in T1 CRC in an independent clinical cohort and development of risk-stratification model

Next, to further confirm the performance of our cf- and exo-miRNA combination panel, we evaluated its performance in an independent and larger cohort of patients with T1 CRC. To demonstrate the rigor and reproducibility of the regression analysis developed in the training cohort, we applied the exact statistical correlates in an independent validation cohort comprising 142 CRC patients. It was exciting to note that even in this cohort, our panel performed robustly in identifying patients (AUC; 0.84, PPV; 0.33, Supplemental Fig. 2), highlighting its clinical significance for detecting the presence of LNM in patients with T1 CRC.

**Fig. 2:**
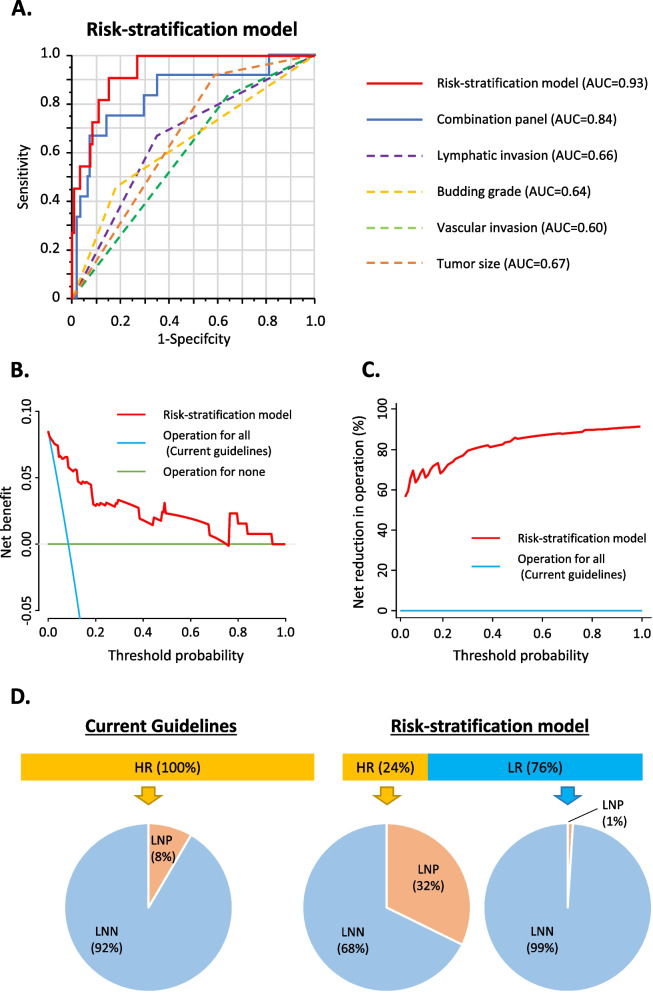
Performance evaluation of novel combination panel and risk-stratification model in predicting lymph node metastasis from high-risk T1 CRC patients. **A** A receiver operating characteristic curve analysis to compare the performance of the combination panel, key pathological features, and risk-stratification model in a validation cohort. **B** Decision curve plotting net benefit (detection of lymph node metastasis) against threshold probability. **C** Decision curve plotting net benefit untreated (avoidance of unnecessary operation) against threshold probability. **D** Comparison of overtreatment frequency between current guidelines and our risk-stratification model. NPV: negative predictive value, PPV: positive predictive value, LNN: lymph node metastasis negative, LNP: lymph node metastasis positive, AUC: area under the curve

Next, we compared the performance of this combination panel with other key pathological features currently used in clinical settings, including lymphatic invasion, tumor budding grade, vascular invasion, and tumor size (Fig. 2A). Even in these analyses, our exosome-based transcriptomic panel exhibited higher AUC and PPV (AUC; 0.84, PPV; 0.33) compared to all pathological features, including lymphatic invasion (AUC; 0.66, PPV; 0.15), tumor budding grade (AUC; 0.64, PPV; 0.19), vascular invasion (AUC; 0.60, PPV; 0.11), and tumor size (AUC; 0.67, PPV; 0.13). We next asked whether there was any additive value in combining our molecular markers with key pathological features. When we developed such a risk-stratification model by adding pathological features to our combination panel, we noticed a significant improvement in the performance of this model for its ability to detect LNM in patients with T1 CRC (AUC; 0.93, PPV; 0.36, Fig. 2A).

We performed univariate and multivariate analyses to confirm which biomarkers have the highest ability to detect LNM (Supplemental Table 2). In univariate analysis, while high tumor budding grade (OR: 3.889, *p* = 0.04) and lymphatic invasion (OR: 3.778, p = 0.04) predicted LNM, our combination panel (OR; 18.667, *p* < 0.01) and risk-stratification model (OR; 56.111, p < 0.01) were superior predictors of LNM given the significantly higher ORs vs. pathological factors. Moreover, in the multivariate analysis, only our risk-stratification model (OR; 14.66, 95% CI; 1.0299–209.43) emerged as an independent predictor of LNM in patients with high-risk T1 CRC (Supplemental Table 2), once again highlighting the clinical usefulness of our cf- and exo-miRNAs combination panel-based risk-stratification model for selection of patients that are LNM-positive and require radical surgery.

### The risk-stratification model allows a more prudent clinical decision-making and selection of T1 CRC patients with LNM and significantly reduce the overtreatment burden

Moreover, we performed a decision curve analysis (DCA) to clarify the clinical usefulness of our risk-stratification model. First, we derived a decision curve for LNM detection in high-risk T1 CRC patients (Fig. 2B). As illustrated in the DCA, the green line indicates an operation for none of the patients. In contrast, the blue line indicates an operation for all patients – which will be the recommendation with the current guidelines for all high-risk patients. In contrast, our risk-stratification model demonstrated a significantly higher net benefit of surgery only in true LNP patients (red line) vs. the current guidelines in all threshold probabilities, highlighting its clinical significance for identifying true high-risk T1 CRC patients with LNM. Second, this DCA plot also highlights patients that can avoid unnecessary operations (Fig. 2C). Our risk-stratification model showed a relatively high percentage of unnecessary operation reduction, even at the smallest threshold probability. From a clinical standpoint, a small threshold probability indicates that missing a patient with LNM is more critical than reducing unnecessary operations in this setting. Therefore, our risk-stratification model can help reduce a significant percentage of unnecessary operations without missing any cases with LNM. These findings highlight that our risk-stratification model is superior to current guidelines for LNM detection and avoiding unnecessary operation.

According to current guidelines, 92% of patients within our validation cohort underwent unnecessary operations (Fig. 2D left panel). In contrast, when we applied our optimized risk-stratification model to the patients within the same cohort, we significantly reduced the frequency of radical surgery in 76% of patients while missing less than 1% of patients who were LNP (Fig. 2D right panel). Moreover, the percentages of LNP among high-risk patients were 4 times higher in the risk-stratification model compared to the current guidelines. In summary, our newly developed risk-stratification model can robustly risk stratifying patients with T1 CRC who genuinely have LNM and require radical surgery, spare the test for overtreatments, and reduce healthcare costs associated with such surgical procedures.

In the present study, we identified a panel of 4 miRNAs, including miR-181b, miR-193b, miR-195, and miR-411, within the exosomal component with promising biomarker potential in pre-operative serum for the prediction of lymph node metastasis (LNM) among patients with T1 CRC. Subsequently, we demonstrate that exosomal miRNAs (exo-miRNAs) are superior cancer biomarkers compared to cell-free miRNAs (cf-miRNAs). Still, more importantly, a combination of cf- and exo-miRNAs are superior to individual biomarker panels. Finally, we validated this combination biomarker panel in an independent clinical cohort. We developed a risk-stratification model by including key pathological features currently used in the clinic for detecting LNM, which further improved the overall diagnostic accuracy for identifying true high-risk T1 CRC patients.

We would like to acknowledge a potential limitation of our study. Both clinical cohorts analyzed for biomarker training and validation in this retrospective study were relatively modest in size and were of Japanese heritage. Hence, a future prospective study with larger patient cohorts from multinational cohorts might be necessary to confirm our biomarkers’ clinical significance further.

## Conclusions

In conclusion, our newly developed risk-stratification model can robustly stratify T1 CRC patients into true high-risk and low-risk groups for the presence of LNM, which could help in a more informed clinical decision-making for selecting appropriate patient subsets that require radical operation and spare the rest from overtreatment and reduce associated healthcare costs associated with such surgeries.

## Supplementary Information

**Additional file 1.** Materials and methods.

**Additional file 2: Supplemental Table 1.** Clinicopathological factors of clinical cohorts.

**Additional file 3: Supplemental Table 2.** Univariate and multivariate analysis for lymph node metastasis detection in the validation cohort.

**Additional file 4: Supplemental Figure 1.** Comparison of diagnostic ability of four cell-free miRNAs and four exosomal miRNAs in lymph node metastasis detection. A) A receiver operating characteristic (ROC) curve analysis to evaluate the performance of four cell-free miRNAs. B) ROC curve analysis to evaluate the performance of four exosomal miRNAs. Cf-: cell-free, exo: exosomal, AUC: area under the curve.

**Additional file 5: Supplemental Figure 2.** Validation of novel combination panel in predicting lymph node metastasis from high-risk T1 CRC patients. Left chart) A receiver operating characteristic curve analysis to evaluate the performance of exosomal and cell-free miRNAs combination panel in a validation cohort. Right chart) Waterfall plot for modified risk score distribution in a validation cohort. LNN: lymph node metastasis negative, LNP: lymph node metastasis positive, AUC: area under the curve.

**Additional file 6: Supplemental Figure 3.** A nomogram illustrates the probability of lymph node metastasis risk.

## Data Availability

All other data are available on reasonable request from the corresponding authors.

## Abbreviations

AUC: Area under the curve
Cf-miRNAs: cell-free miRNAs
CRC: Colorectal cancer
DCA: Decision curve analysis
EMR: Endoscopic mucosal resection
ESD: Endoscopic submucosal dissection
Exo-miRNAs: Exosomal miRNAs
LNM: Lymph node metastasis
LNN: Lymph node metastasis-negative
LNP: Lymph node metastasis-positive
miRNA: Micro RNA
NPV: Negative predictive value
OR: Odds ratio
PPV: Positive predictive value
RT-qPCR: Real-time quantitative reverse transcription polymerase chain reaction
ROC: Receiver operator characteristics
T1 CRC: Invasive submucosal colorectal cancer
