# Supplementary material for: An exosome-based liquid biopsy signature for pre-operative identification of lymph node metastasis in patients with pathological high-risk T1 colorectal cancer
Source: Mol Cancer. 2023 Jan 6;22:2. doi: 10.1186/s12943-022-01685-8 (PMC9817247; doi:10.1186/s12943-022-01685-8)
Supplement: Supplementary file 1 — Additional file 1. Materials and methods. [file 12943_2022_1685_MOESM1_ESM.docx]

**MATERIALS AND METHODS**

Patient cohorts

This retrospective cohort study included a total of 200 high-risk T1 CRC patients. All patients were pathologically characterized as high-risk for LNM and were subjected to radical surgery. These patients were enrolled at 2 independent institutions. The first cohort was used for biomarker training and included 58 patients with 7 who were LNM-positive (LNP) and 51 LNM-negative (LNN) – enrolled at the Tokyo Medical and Dental University Hospital, Japan. The second cohort was assigned as the validation cohort and comprised of 142 patients with 12 LNP and 130 LNN, who were enrolled at the National Cancer Center Hospital East, Japan. Radical surgeries were performed during the period between January 2012 and November 2014 in the training cohort, and between January 2011 and December 2017 in the validation cohort. Pre-operative serum samples were obtained from these patients and were used for analysis in this study.

Pathological high-risk LNM patients were diagnosed according to Japanese Society for Cancer of the Colon and Rectum guidelines 2019 for the treatment of colorectal cancer; pT1b (depth of submucosal invasion > 1000 μm), lymphovascular invasion positive, histology (poorly differentiated adenocarcinoma, signet-ring carcinoma, or mucinous carcinoma), and high grade of tumor budding at the site of deepest invasion ([1](#_ENREF_1)). Radical surgery was performed by standard procedure which included intestinal resection with lymph node dissection. All surgical specimens were evaluated by pathologists at each participating institution and pathologically confirmed for the presence or absence of LNM. We excluded all patients with synchronous advanced CRC, and those with distant metastasis or non-adenocarcinoma.

This study was approved by ethics review committee of each participating institution, and all biospecimen collection and subsequent laboratory analyses were performed in accordance with the Declaration of Helsinki. All patients provided a written informed consent for their participation in this study.

Exosome isolation from serum specimens

Total exosome isolation was performed by using Total Exosome Isolation Kit (from serum) according to manufacturer’s recommendation protocol (ThermoFisher Scientific, Waltham, MA, USA). Briefly, 200 μL of serum was thawed on ice and centrifuged at 2000 ×g for 30 min at room temperature to remove cells and debris. Afterwards, we added 40 μL of the Total Exosome Isolation reagent and incubated the contents for 30 min at 4^o^C. Following incubation, samples were centrifuged at 10,000 ×g for 10 min at room temperature and the supernatant was discarded. Exosomes were contained in the pellet, which were resuspended using 200 μL of phosphate-buffered saline (PBS). Isolated exosomes were preserved at 4^o^C until RNA extraction.

RNA extraction from serum and exosomes

The procedure for RNA extraction from serum was as described previously by Wada and colleagues ([2](#_ENREF_2)). Total RNA extraction was performed using the Qiagen miRNeasy Kit (Qiagen, Hilden, Germany). Briefly, 200 μL of samples were lysed in 1000 μL of Qiazol solution. Thereafter, by using QIAcube Automated System (Qiagen, Hilden, Germany), total RNA was subsequently extracted and purified. Extracted RNA was subsequently converted into complementary DNA (cDNA) prior to polymerase chain reaction (PCR) assays. The cDNA conversion was performed using the TaqMan microRNA Reverse Transcription Kit (ThermoFisher Scientific, Waltham, MA, USA). The procedure for RNA extraction from exosomes was same as for serum.

Real-time quantitative reverse transcription PCR (RT-qPCR) assays

The RT-qPCR assays were performed as described previously ([2](#_ENREF_2)). Briefly, the following probes were used for TaqMan miRNA assays (Thermo Fisher Scientific, Inc., Waltham, MA, USA): hsa-miR-181b (ID, 001098), hsa-miR-193b-3p (ID, 002367), hsa-miR-195-5p (ID, 000494), hsa-miR-411-5p (ID, 001610), and hsa-miR-16 (ID, 000391). Real-time reverse transcription quantitative PCR analysis was performed by using the QuantStudio 7 Flex Real Time PCR System (Applied Biosystems, Foster City, CA) and the expression of the target miRNAs was normalized to that of miR-16.

Statistical analysis

All statistical analyses were performed by using JMP 8.0.1 (SAS Institute Inc., Cary, NC), EZR 1.54 (Saitama Medical Center, Jichi Medical University, Saitama, Japan), and R (4.0.3, R Development Core Team, https://cran.r-project.org/). In all tests, P values less than 0.05 were considered as statistically significant. The correlation of each miRNA expression in exosomes and cell-free component was evaluated by Pearson’s correlation coefficients (r). Receiver operator characteristic (ROC) curves were used to evaluate the ability of individual miRNAs and biomarker panels for detecting LNM detection. The cut-off thresholds were determined using Youden’s index. Binary logistic regression models were used to train a classifier based on the expression of exo-miRNAs and cf-miRNAs. Once the model was established in the training cohort, the same statistical covariates were subsequently applied in the validation cohort. The decision curve analysis was performed by using the ‘devtools’ and ‘DCA’ package in R ([3-5](#_ENREF_3)).

**References**

1. Hashiguchi Y, Muro K, Saito Y, Ito Y, Ajioka Y, Hamaguchi T, et al. Japanese Society for Cancer of the Colon and Rectum (JSCCR) guidelines 2019 for the treatment of colorectal cancer. International journal of clinical oncology. 2020;25(1):1-42.

2. Wada Y, Shimada M, Murano T, Takamaru H, Morine Y, Ikemoto T, et al. A Liquid Biopsy Assay for Noninvasive Identification of Lymph Node Metastases in T1 Colorectal Cancer. Gastroenterology. 2021;161(1):151-62.e1.

3. Zhang Z, Rousson V, Lee WC, Ferdynus C, Chen M, Qian X, et al. Decision curve analysis: a technical note. Annals of translational medicine. 2018;6(15):308.

4. Rousson V, Zumbrunn T. Decision curve analysis revisited: overall net benefit, relationships to ROC curve analysis, and application to case-control studies. BMC medical informatics and decision making. 2011;11:45.

5. Deniffel D, Abraham N, Namdar K, Dong X, Salinas E, Milot L, et al. Using decision curve analysis to benchmark performance of a magnetic resonance imaging-based deep learning model for prostate cancer risk assessment. European radiology. 2020;30(12):6867-76.
