# Supplementary material for: An exosome-based liquid biopsy signature for pre-operative identification of lymph node metastasis in patients with pathological high-risk T1 colorectal cancer
Source: Mol Cancer. 2023 Jan 6;22:2. doi: 10.1186/s12943-022-01685-8 (PMC9817247; doi:10.1186/s12943-022-01685-8)
Supplement: Supplementary file 2 — Additional file 2: Supplemental Table 1. Clinicopathological factors of clinical cohorts. [file 12943_2022_1685_MOESM2_ESM.docx]

| **Supplemental Table 1:** Clinicopathological factors of clinical cohorts   \| Characteristics \| Training cohort  (n=58) \| Validation cohort  (n=142) \| p-value \| \| --- \| --- \| --- \| --- \| \| Age ,y  Median (range) \| 69.5 (31-86) \| 67 (24-85) \| 0.02 \| \| Gender  Male  Female \| 34 (59)  24 (41) \| 86 (61)  56 (39) \| 0.80 \| \| LNM  Positive  Negative \| 7 (12)  51 (88) \| 12 (8)  130 (92) \| 0.43 \| \| MSI status  MSI-H  MSI-L  MSS \| N/A  N/A  N/A \| 10 (7)  5 (4)  127 (89) \| NA \| \| Tumor location  Right side  Left side \| 23 (40)  35 (60) \| 41 (29)  101 (71) \| 0.14 \| \| Tumor size, mm  ≥20  <20 \| 21 (36)  37 (64) \| 55 (39)  87 (61) \| 0.74 \| \| Submucosal invasion, µm  ≥1000  <1000 \| 39 (67)  19 (33) \| 140 (99)  2 (1) \| < 0.01 \| \| Budding grade  ≥2  1  Unavailable \| N/A  N/A  N/A \| 26 (18)  104 (74)  12 (8) \| NA \| \| Lymph invasion  Positive  Negative \| 15 (26)  43 (74) \| 53 (37)  89 (63) \| 0.12 \| \| Vascular invasion  Positive  Negative \| 21 (36)  37 (64) \| 49 (35)  93 (65) \| 0.82 \| \| Differentiation  Well / Moderate  Poor \| 56 (97)  2 (3) \| 140 (99)  2 (1) \| 0.35 \|   NOTE. Data are shown as n (%) unless indicated otherwise.  MSI, microsatellite instability; MSI-H, high-frequency microsatellite instability; MSI-L, low-frequency microsatellite instability; MSS, microsatellite stable. |  |
| --- | --- | --- | --- | --- | --- | --- | --- | --- | --- | --- | --- | --- | --- | --- | --- | --- | --- | --- | --- | --- | --- | --- | --- | --- | --- | --- | --- | --- | --- | --- | --- | --- | --- | --- | --- | --- | --- | --- | --- | --- | --- | --- | --- | --- | --- | --- | --- | --- | --- |
