# Supplementary material for: An exosome-based liquid biopsy signature for pre-operative identification of lymph node metastasis in patients with pathological high-risk T1 colorectal cancer
Source: Mol Cancer. 2023 Jan 6;22:2. doi: 10.1186/s12943-022-01685-8 (PMC9817247; doi:10.1186/s12943-022-01685-8)
Supplement: Supplementary file 3 — Additional file 3: Supplemental Table 2. Univariate and multivariate analysis for lymph node metastasis detection in the validation cohort. [file 12943_2022_1685_MOESM3_ESM.docx]

**Supplemental Table 2:** Univariate and multivariate analysis for lymph node metastasis detection in the validation cohort

|  | Univariate analysis | | | Multivariate analysis | | |
| --- | --- | --- | --- | --- | --- | --- |
|  | OR | 95% CI | *P*-value | OR | 95% CI | *P*-value |
| Age  (≥65 y / <65 y) | 0.73 | 0.22 – 2.40 | 0.61 | 0.60 | 0.11 – 3.38 | 0.57 |
| Gender  (Male / Female) | 1.33 | 0.38 – 4.66 | 0.65 | 1.95 | 0.37 – 10.38 | 0.43 |
| Tumor location  (Right / Left) | 0.81 | 0.21 – 3.15 | 0.76 |  |  |  |
| Tumor size, mm  (≥20 mm / <20 mm) | 7.82 | 0.98 – 62.35 | 0.05 | 3.05 | 0.17 – 54.87 | 0.45 |
| Submucosal invasion  (≥1000 µm / <1000µm) | <0.01 |  | 0.99 |  |  |  |
| Budding grade  (≥2 / 1) | 3.89 | 1.09 – 13.95 | **0.04** | 3.81 | 0.61 – 23.84 | 0.15 |
| Lymphatic invasion  (Positive / Negative) | 3.78 | 1.08 – 13.23 | **0.04** | 1.36 | 0.19 – 9.67 | 0.76 |
| Vascular invasion  (Positive / Negative) | 0.35 | 0.04 – 1.68 | 0.19 |  |  |  |
| Differentiation  (Well-Moderate / Poor) | <0.01 |  | 0.99 |  |  |  |
| MSI status  (MSI-H / MSI-L, MSS) | 1.22 | 0.14 – 10.56 | 0.86 |  |  |  |
| Combination panel  (High risk / Low risk) | 18.67 | 4.61 – 75.56 | **<0.01** | 5.66 | 0.86 – 37.08 | 0.07 |
| Risk-stratification model  (High risk / Low risk) | 56.11 | 6.76 – 465.56 | **<0.01** | 14.66 | 1.02 – 209.43 | **< 0.05** |

NOTE. Data are shown as n (%) unless indicated otherwise. The values in bold letters indicate statistical significance.

MSI, microsatellite instability; MSI-H, high-frequency microsatellite instability; MSI-L, low-frequency microsatellite instability; MSS, microsatellite stable.
