# Supplementary figures and images for: An exosome-based liquid biopsy signature for pre-operative identification of lymph node metastasis in patients with pathological high-risk T1 colorectal cancer
Source: Mol Cancer. 2023 Jan 6;22:2. doi: 10.1186/s12943-022-01685-8 (PMC9817247; doi:10.1186/s12943-022-01685-8)

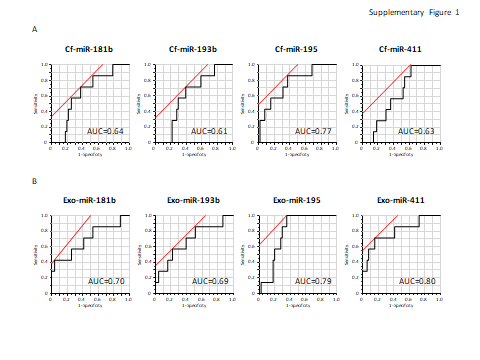

Supplement: Supplementary file 4 — Additional file 4: Supplemental Figure 1. Comparison of diagnostic ability of four cell-free miRNAs and four exosomal miRNAs in lymph node metastasis detection. A) A receiver operating characteristic (ROC) curve analysis to evaluate the performance of four cell-free miRNAs. B) ROC curve analysis to evaluate the performance of four exosomal miRNAs. Cf-: cell-free, exo: exosomal, AUC: area under the curve. [file 12943_2022_1685_MOESM4_ESM.docx]

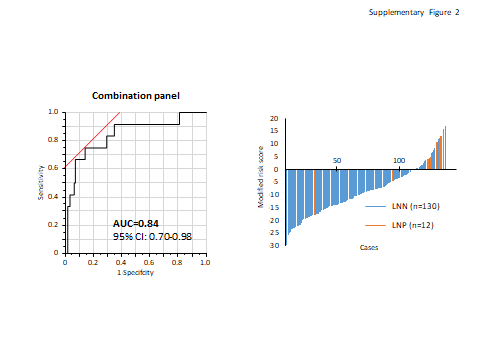

Supplement: Supplementary file 5 — Additional file 5: Supplemental Figure 2. Validation of novel combination panel in predicting lymph node metastasis from high-risk T1 CRC patients. Left chart) A receiver operating characteristic curve analysis to evaluate the performance of exosomal and cell-free miRNAs combination panel in a validation cohort. Right chart) Waterfall plot for modified risk score distribution in a validation cohort. LNN: lymph node metastasis negative, LNP: lymph node metastasis positive, AUC: area under the curve. [file 12943_2022_1685_MOESM5_ESM.docx]

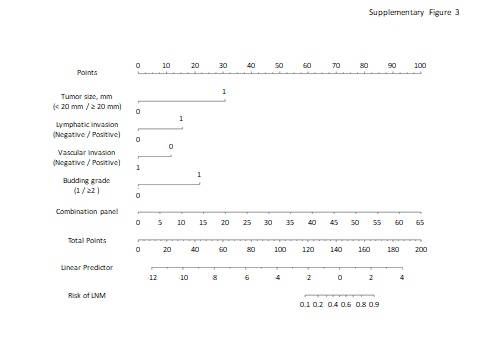

Supplement: Supplementary file 6 — Additional file 6: Supplemental Figure 3. A nomogram illustrates the probability of lymph node metastasis risk. [file 12943_2022_1685_MOESM6_ESM.docx]
